# Alternative signal priming enhances the inflammatory response in macrophages

**DOI:** 10.1038/s41423-023-00984-4

**Published:** 2023-02-17

**Authors:** Huihao Tang, Xiaojie Li, Taomin Huang, Xiaolei Ding

**Affiliations:** 1 Institute of Geriatrics, Affiliated Nantong Hospital of Shanghai University (The Sixth People’s Hospital of Nantong), School of Medicine, Shanghai University, Nantong, 226011 China; 2 Shanghai Engineering Research Center of Organ Repair, School of Medicine, Shanghai University, Shanghai, 200444 China; 3 Department of Pharmacy, Eye & ENT Hospital, Fudan University, Shanghai, 200031 China

**Keywords:** Monocytes and macrophages, Inflammation

A recent study by Czimmerer et al. published in *Immunity* reveals that exposure to interleukin-4 (IL-4), which is an alternative signal, remodels the macrophage epigenetic landscape, including chromatin structure and the binding of transcriptional regulators to their dedicated enhancers. This allows for a synergistic interaction with toll-like receptor (TLR) ligand-mediated inflammatory stimuli, consequently enabling these cells to have an exaggerated inflammatory response [1].

**Fig. 1:**
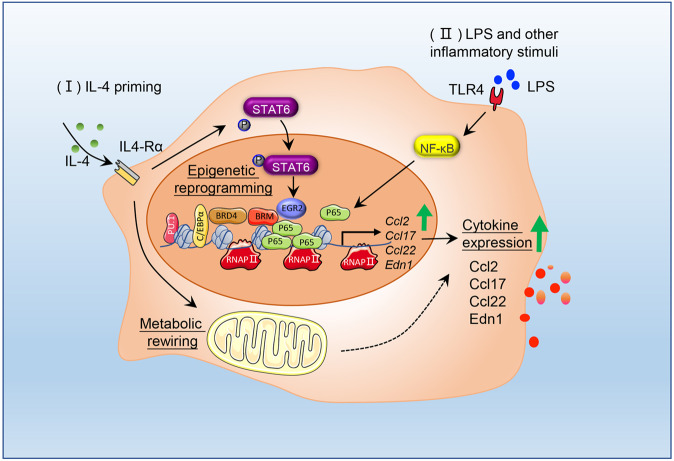
IL-4 exposure triggers an enhanced inflammatory response in macrophages. Mouse bone marrow-derived macrophage (BMDM) identity is maintained by PU.1 and C/EBPα, two major myeloid lineage determining factors. Exposure of BMDMs to IL-4 (I) activates STAT6 through phosphorylation, which leads to chromatin landscape remodeling at specific genomic loci via STAT6-EGR2 signaling. Enhancers at transcriptionally active loci show increased chromatin accessibility and binding with NF-κB-p65, which enhance TLR ligand-mediated inflammatory activation (II). In addition, IL-4 signaling induces macrophage metabolic rewiring, which contributes to the subsequent inflammatory response, as described by Lundahl et al. [8]

Macrophages are a heterogeneous population of innate immune cells that have an extraordinary capacity to adapt to their surroundings in response to various signals. Appropriate activation is crucial for proper host defense against infection, debris clearance, and efficient tissue repair, whereas aberrant activation is closely associated with tumorigenesis, metabolic disorders, and impaired tissue repair [2]. The molecular signatures of classic and alternative macrophage activation have been well characterized [3]. Over the past several years, increasing evidence has highlighted that macrophages can sense inflammatory stimuli and alter their subsequent behaviors (referred to as “innate training”) [4]. However, there is limited understanding of how activation signals interact synergistically or antagonistically to determine macrophage activation and functional status. Moreover, tissue-resident macrophage activation is complex and regulated in a context-specific manner, which is a challenge in determining their functions and phenotypes in vivo. Further insights into the molecular basis of the regulatory networks of macrophage activation might ultimately enable the development of new therapies to treat inflammatory diseases and promote tissue repair. Czimmerer et al. presented mechanistic data that alternative signaling epigenetically reprograms macrophages, which drives an enhanced inflammatory response (Fig. 1).

Using mouse bone marrow-derived macrophages (BMDMs), Czimmerer et al. examined whether alternative signal priming affected subsequent inflammatory stimuli. Transcriptomic analyses revealed a significant transcriptional difference with and without IL-4 exposure when the TLR agonist lipopolysaccharide (LPS) was added. Specifically, a group of proinflammatory genes (e.g., *Ccl2*, *Ccl17*, and *Ccl22*) was significantly upregulated (Fig. 1). The NF-κB transcription factor complex is a major regulator of the macrophage inflammatory response. Surprisingly, neither NF-κB expression nor its cellular location was altered by IL-4 exposure. However, chromatin immunoprecipitation and sequencing (ChIP-Seq) analyses identified that IL-4-exposed cells exhibited an expansion of the LPS-induced NF-κB-p65 cistrome. Transcription factors require chromatin opening to bind to their target DNA motif, which is controlled by epigenetic mechanisms. Next, the authors performed a series of elegant experiments, including assays for transposase-accessible chromatin with sequencing (ATAC-Seq) and computational analysis, and found that IL-4 exposure remodeled macrophage epigenetic profiles. The altered chromatin allowed the components of RNA polymerase and NF-κB-p65 to access a repertoire of active enhancers, leading to a more pronounced inflammatory response (Fig. 1). The importance of chromatin remodeling in IL-4 signaling-mediated synergistic effects was further demonstrated by the investigation of epigenetic modifiers, and inhibiting the epigenetic modifier BRD4 reduced the extended synergy.

Signal transducer and activator of transcription 6 (STAT6) is a major effector that mediates IL-4 responses in macrophages. Transient activation of STAT6 induces EGR2, which is a transcription factor that controls late transcriptional programs associated with alternative polarization [5]. Concomitantly, in STAT6- and EGR2-deficient BMDMs, the synergistic effects of IL-4 exposure were decreased. ATAC-seq analysis indicated that chromatin openness and transcriptional regulator binding triggered by IL-4 exposure occurred in an EGR2-dependent fashion. Thus, EGR2 binds to its response element and mediates IL-4 exposure-induced epigenomic remodeling.

The authors further tested whether these synergistic effect occurred in organismal physiology and pathology using a mouse model of pulmonary allergic airway inflammation. In this mouse model, an expanded pool of alternatively activated macrophages was present in the airways. Indeed, the mRNA and protein levels of synergistically induced genes were markedly induced in macrophages isolated from alveolar tissue during allergic airway inflammation compared with those from healthy controls, suggesting that alternative activation of alveolar macrophages contributes to an extended synergy in the inflammatory response.

Although it is becoming increasingly clear that epigenetic modulation is crucial for macrophage phenotypic switching, the study by Czimmerer et al. is the first to provide a mechanistic understanding at multiple molecular levels by which IL-4 signal priming imprints macrophages and facilitates the subsequent inflammatory response. This study raises several exciting questions for further investigation. First, macrophages can undergo multiple alternative activation states in response to various stimuli, such as transforming growth factor β (TGFβ), interleukin 10 (IL-10), and chitin [6]. The next question is whether such a synergistic effect occurs in general or is restricted to IL-4-induced alternative activation. Second, macrophage metabolism affects their activation and function. In particular, the acquisition of an alternative phenotype is accompanied by a dramatic shift in fatty acid oxidation and oxidative phosphorylation (OXPHOS) metabolism [7]. Interestingly, another recent study published by Lundahl et al. in *eLife* in 2022 had a very similar experimental approach and reported that IL-4 and interleukin 13 (IL-13) exposure induced BMDM innate training that enhanced proinflammatory and bactericidal responses against mycobacteria [8]. According to Lundahl et al., IL-4 and IL-13 training triggers OXPHOS, which is necessary for the subsequent enhanced inflammatory response. Thus, a key question is how changes in epigenetic landscapes and cellular metabolism interact reciprocally to drive the extended synergy or innate training described above. Third, a similar observation in human macrophages is encouraging. Allergic asthma is a typical type 2 inflammatory condition with increased numbers of alternatively activated macrophages. Intriguingly, a recent study showed that asthma patient-derived macrophages exhibited inflammatory memory [9]. Whether human macrophages exhibit similar epigenetic and transcriptional mechanisms merits further investigation.

Finally, what are the functional consequences of macrophage induction of a hyperinflammatory gene expression program? Several of the synergistically upregulated genes described here, including *Ccl2*, *Ccl17* and *Ccl22*, are best known for their roles in regulating the recruitment of neutrophils, macrophages, and other immune cells. It is possible that improved recruitment results in the rapid clearance of bacteria, as described by Lundahl et al. Dynamic and overlapping phenotypes of macrophage activation have been described in different scenarios, such as tissue repair, tumorigenesis, therapeutic interventions, and metabolic diseases [10, 11]. Precisely determining the functional role of extended synergy in vivo is complex and challenging. Further research into the mechanisms of macrophage activation using new techniques, such as single-cell analysis and lineage tracing, will not only provide more insights into the functional contributions of specific macrophage subsets in health and disease but also allow us to avoid excessive inflammatory responses and enhance regeneration after injury by tailoring their activation.
